# Functional Amyloids in Reproduction

**DOI:** 10.3390/biom7030046

**Published:** 2017-06-29

**Authors:** Aveline Hewetson, Hoa Quynh Do, Caitlyn Myers, Archana Muthusubramanian, Roger Bryan Sutton, Benjamin J. Wylie, Gail A. Cornwall

**Affiliations:** 1 Department of Cell Biology and Biochemistry, Texas Tech University Health Sciences Center, Lubbock, TX 79430, USA; aveline.hewetson@ttuhsc.edu (A.H.); quynh-hoa.do@ttuhsc.edu (H.Q.D.); caitlyn.myers@ttuhsc.edu (C.M.); archana.muthusubramanian@ttuhsc.edu (A.M.); 2 Department of Cell Physiology and Molecular Biophysics, Texas Tech University Health Sciences Center, Lubbock, TX 79430, USA; roger.b.sutton@ttuhsc.edu; 3 Department of Chemistry, Texas Tech University, Lubbock, TX 79409, USA; benjamin.j.wylie@ttu.edu

**Keywords:** amyloid, reproduction, spermatogenesis, fertilization, epididymis, zona pellucida, semen, cystatins, germline specification

## Abstract

Amyloids are traditionally considered pathological protein aggregates that play causative roles in neurodegenerative disease, diabetes and prionopathies. However, increasing evidence indicates that in many biological systems nonpathological amyloids are formed for functional purposes. In this review, we will specifically describe amyloids that carry out biological roles in sexual reproduction including the processes of gametogenesis, germline specification, sperm maturation and fertilization. Several of these functional amyloids are evolutionarily conserved across several taxa, including human, emphasizing the critical role amyloids perform in reproduction. Evidence will also be presented suggesting that, if altered, some functional amyloids may become pathological.

## 1. Introduction

Amyloids are proteins that self-assemble into highly ordered cross-β-sheet rich structures that typically have been viewed as pathological entities associated with neurodegenerative disease and prionopathies. Growing evidence, however, indicates that most proteins can adopt the amyloid fold given the correct environment and that some amyloids carry out nonpathological biological roles and are known as functional amyloids [1]. Indeed, the amyloid fold is ancient and believed to represent a biological structure of early life [2]. In a test of whether amyloid functioned as a prebiotic entity, Greenwald and coworkers demonstrated that alanine and valine, thought to be abundant on prebiotic earth, polymerized into peptides and cross-β-sheet amyloid fibrils following exposure to carbonyl sulfide, a volcanic gas [3]. The now-recognized affinity of amyloids for nucleic acids and membranes/lipids further supports their role in early life.

Considering that, once formed, perpetuation of life must also occur, it is not surprising that several of the most fundamental processes in reproduction, including gamete formation and gamete interactions, utilize functional amyloids and that their use has been conserved throughout evolution. In some cases, in spite of a divergence in amino acid sequence of proteins involved in these reproductive processes, their propensity to form amyloid has been maintained allowing conservation of the structure across species. In this review, we will specifically discuss amyloids that perform biological roles in reproduction, properties that may distinguish them from pathological amyloids, and evidence suggesting that under some conditions reproductive functional amyloids may become pathological. The amyloids we will describe herein range from those consisting seemingly of individual proteins functioning in their amyloid state to complex functional amyloid matrices composed of several to many proteins.

## 2. Gametogenesis

Sexual reproduction requires the production of haploid germ cells from diploid progenitor cells by the process of meiosis. A number of RNA binding proteins mediate this process, including controlling the translation of cyclins which direct progression through the meiotic divisions [4]. Although budding yeast normally reproduce asexually, under some conditions, including nutrient starvation, they produce spores or germ cells that fuse with cells of an opposite mating type in sexual reproduction. Berchowitz et al. 2015 showed that the sporulation-specific RNA binding protein Rim4 forms amyloid in yeast during sexual reproduction [5]. The Rim4 amyloid binds to the 5′ untranslated region (UTR) of the cyclin B-type cyclin 3 (CLB3) mRNA inhibiting its translation and allowing homologous chromosome segregation in meiosis I. Nearing the end of meiosis I, Rim4 amyloid is degraded allowing the CLB3 mRNA to be translated and cells to complete meiosis II [5]. If CLB3 is translated earlier during meiosis I, cells undergo a division that is more like mitosis than meiosis demonstrating the importance of translational control during gametogenesis and the role of amyloids in mediating this process [6]. Rim4 is a 71 kDa protein which contains a low-complexity/disordered region with a poly-N-rich domain, hallmarks of prions; further, deletion of the low-complexity region prevents Rim4 amyloid formation and its ability to repress translation [5,7]. Rim4 function is not unlike Sup35, a well-characterized yeast prion, that normally functions as a translation termination factor, but in its amyloid state allows translational read-through resulting in divergent phenotypes [8]. However, whether Rim4 is a prion and exhibits non-genetic inheritance has not been determined.

Translational control of critical mRNAs is also needed during spermatogenesis in mice and human. Deleted in AZoospermia-like (DAZL), an RNA binding protein that mediates translation in mice and which is essential for gametogenesis, and the related human meiosis-specific DAZ1-DAZ4 proteins, which have predicted prion-like domains, are proposed to function much like Rim4 in yeast [9,10,11]. Although the amyloid properties of DAZL/DAZ have not been established, the presence in germ cells of DAZL in sodium dodecyl sulfate (SDS)-resistant aggregates, consistent with amyloid, suggests that functional amyloids may be an evolutionarily conserved mechanism to control processes integral for gametogenesis [5].

## 3. Germline Specification

In many sexually reproducing organisms, embryonic cells differentiate giving rise to somatic tissues as well as maintaining a germline that will generate spermatozoa and oocytes. In oocytes of several species, germline components are assembled into a nonmembrane-bound structure in the cytoplasm known as a Balbiani body [12]. Since oocytes can be dormant for long periods of time prior to activation before fertilization, the Balbiani bodies may protect the specific maternal RNAs and organelles required during embryogenesis [13,14]. Studies in *Xenopus laevis* revealed that Xvelo, a disordered protein with a predicted prion-like domain, is a major component of Balbiani bodies and its assembly into an amyloid matrix via its prion-like domain forms the Balbiani body structure [15]. Furthermore, the Xvelo amyloid matrix binds RNA, mitochondria and other organelles sequestering them within the Balbiani body for storage. Xvelo is also present in mature eggs but not as an amyloid, suggesting that it disaggregates during development [15].

In zebrafish, Bucky ball is essential for Balbiani body formation [16]. Despite poor sequence similarity between Bucky ball and Xvelo, Bucky ball also has a predicted prion-like domain, suggesting that the propensity to form amyloid is conserved [15]. Similarly, in *Drosophila*, Oskar plays an important role in organization of pole plasm and also is a disordered protein with a predicted prion-like domain [15,17]. In *Caenorhabditis elegans*, germline P granules sequester RNA and protein. While the amyloid properties of P granules have not been determined, they do undergo phase transitions which may represent a primordial mechanism for functional self-assembly [18]. Together these studies show a conservation of amyloidogenic proteins mediating germline specification formation across several species.

## 4. Sperm Acrosome Reaction and Fertilization

Following meiosis and formation of haploid germ cells, male germ cells undergo a metamorphosis called spermiogenesis in which they transition from a round cell to one with a defined head, midpiece and tail. During this process a secretory granule/melanosome-like exocytotic vesicle, known as the acrosome, forms on the surface of the sperm head [19]. In fertilization, the release and/or exposure of the acrosomal contents allows the spermatozoon to penetrate through the investments surrounding the oocyte. The acrosome contains both a soluble fraction and an insoluble acrosomal matrix (AM). The AM is believed to function as a scaffold that mediates the release of AM-associated proteins necessary for fertilization and that interacts with the zona pellucida (ZP) surrounding the oocyte [20,21]. The importance of AM function is emphasized by its conservation in spermatozoa across species including water strider, quail, hamster, guinea pig, bull, stallion, boar, and human, and that sperm lacking acrosomes do not fertilize [22,23].

**Figure 1:**
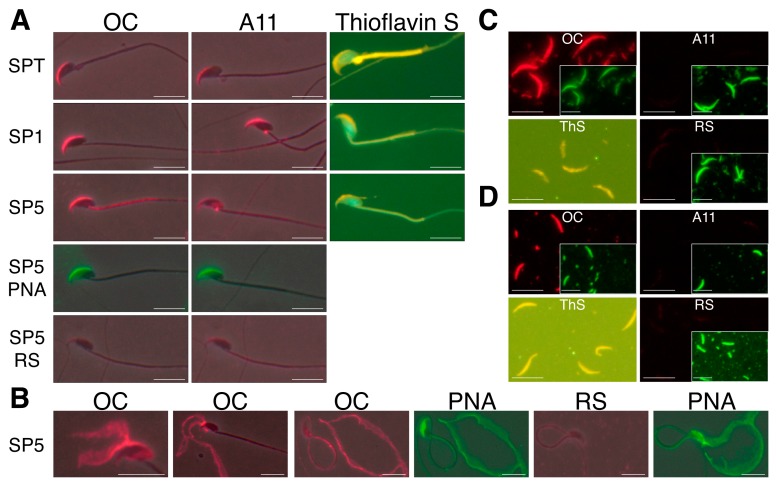
Amyloids are present in mouse sperm acrosomes and isolated AM. Immunofluorescence analysis (IIF) using anti-fibrillar amyloid OC and anti-oligomeric amyloid A11 antiserum and thioflavin S staining showed the presence of amyloid in (**A**) intact acrosomes from testicular (SPT), caput epididymal (SP1), and cauda epididymal spermatozoa (SP5); (**B**) mechanically disrupted acrosomal shrouds from cauda epididymal spermatozoa; and isolated AM from (**C**) caput epididymal and (**D**) cauda epididymal spermatozoa. RS, normal rabbit serum. Staining with fluorescein isothiocyanate peanut agglutinin (FITC-PNA) was used as a marker for acrosomal material. Scale bar, 10 µm; insert, FITC-PNA staining shown at 40% reduction. Reprinted from [24].

We previously demonstrated that amyloids are present within the mouse sperm acrosome and AM and contribute to the formation of an SDS- and formic-acid-resistant core, defined as the scaffold remaining after sequential extraction of isolated AM with 1% SDS and 5% SDS (Figure 1) [24]. Proteomic analyses including liquid chromatography-tandem mass spectrometry (LC–MS/MS) of the isolated AM core revealed a complex structure composed of several known amyloidogenic proteins including cystatin C, cystatin-related epididymal spermatogenic (CRES), and lysozyme; family members of known amyloidogenic proteins such as phosphoglycerate kinase 2 and transglutaminase 3; and proteins with established roles in ZP interaction that were predicted to be amyloidogenic including zonadhesin (ZAN), and zona pellucida 3 receptor (full list of proteins in [24]). Of the fifty-nine proteins identified in the AM core, most were predicted by the Waltz program to have multiple amyloidogenic sites, while only three were predicted to not have any amyloidogenic regions [24]. Although it has yet to be determined if each protein exists as an amyloid conformer in the AM core, our studies suggest that complex functional amyloid matrices composed of many unrelated proteins may be a normal component of biological systems. Rather than all proteins forming the AM amyloid core, it is conceivable that amyloidogenic domains may be a mechanism by which a protein associates with the AM, perhaps as a point of attachment, while maintaining other domains in the soluble, monomeric state.

We also demonstrated that the sperm AM is stable at pH 3 but rapidly disassembles at pH 7. The pH-dependent dispersion of the AM correlates with a change in amyloid structure leading to a loss of mature forms (OC antibody reactive) and a gain of immature amyloid forms (A11 antibody reactive) [24]. This suggests that disaggregation of the AM amyloid may be a critical first step in AM dispersion. The reversal of the AM amyloid raises the possibility that an amyloid disaggregase activity, similar to Hsp104 function in yeast, may be present in the sperm acrosome [25].

The significance of the AM as an amyloid is not known. It is possible that the AM amyloid is important for the sequential release of proteins during the acrosome reaction and/or for proper orientation of signaling complexes that function during fertilization. Spermatozoa from mice lacking the cystatin CRES, an amyloidogenic protein in the AM core, are unable to undergo the acrosome reaction, show reduced protein tyrosine phosphorylation, and exhibit fertility defects in vitro [26]. However, the fertility defect and sperm protein tyrosine phosphorylation, an indicator of sperm capacitation, is rescued by addition of dibutyryl cyclic adenosine monophosphate (cAMP) and the phosphodiesterase inhibitor 3-isobutyl-1-methylxanthine [26]. This suggests that the loss of CRES, and possibly CRES amyloid, from the AM may disrupt key cAMP-dependent signaling cascades necessary for fertilization.

It is also possible that the AM amyloid functions as a defined three-dimensional structure that interacts with the ZP during gamete recognition. Indeed, the model of a single sperm protein functioning as a ligand for the sperm receptor on the oocyte has been replaced by the notion that sperm-ZP binding likely involves a multitude of sperm fertilization molecules [27]. We would add to this hypothesis by suggesting that integration of several or many ZP-binding fertilization molecules into an ordered amyloid matrix might provide the proper three-dimensional orientation for interaction with the three-dimensional ZP matrix. ZAN, which was first identified in sperm based on its binding to the egg ZP, is predicted to have 44 amyloidogenic sites within its sequence and is a part of the AM [28]. Though the significance of these many sites in the large ZAN protein is not known, it is essential for species-specific binding to the ZP as sperm from ZAN knock out (KO) mice are promiscuous and bind to ZP from species other than mice [29]. Despite sperm acrosomes and their AM being highly conserved structures, the amyloid properties of the AM have not been examined in species other than mouse.

## 5. Egg Zona Pellucida and Fertilization

**Figure 2:**
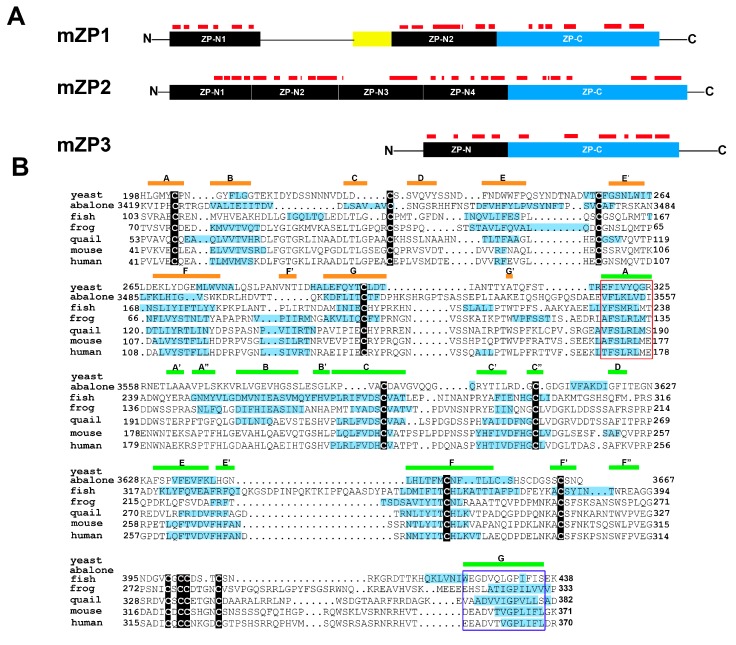
Amyloidogenic sites are present in ZP proteins and are evolutionarily conserved in the zona pellucida (ZP) domain of ZP3 homologs. (**A**) Schematic diagram of mouse ZP1, ZP2, ZP3 with amyloidogenic regions predicted by AmylPred 2 indicated as red bars. Yellow box, trefoil domain. (**B**) Amyloidogenic sites, indicated by blue highlighting, in ZP domains of ZP3 homologs. Modified and reprinted from [41].

The ZP is an extracellular fibrillar matrix that surrounds the oocyte and plays critical roles mediating species-specific gamete recognition and protection from polyspermy. The mouse ZP is formed by three proteins ZP1, ZP2, and ZP3, all of which have a ZP polymerization domain, further divided into N-terminal (ZP-N) and C-terminal (ZP-C) subdomains, that directs fibril formation and assembly into the three-dimensional ZP matrix [30,31] (Figure 2A). Each ZP protein can form homopolymers; however, formation of ZP fibrils requires interaction between ZP3 and ZP1 or ZP2 [32,33]. In addition to the ZP polymerization domain, ZP1 and ZP2 also contain ZP-N repeats in their N-termini which are thought to participate in species-specific interactions with spermatozoa [34]. Egg coats (ZP homologs) surrounding oocytes in nonmammalian vertebrates and invertebrates are also composed of ZP domain-containing proteins that assemble into fibrillar matrices and that function in gamete recognition/interaction [35]. Similarly, the yeast protein α-agglutinin/Sag1p, which is essential for mating by its interactions with a-agglutinin in yeast of opposite mating type, possesses ZP domain-like features [36,37].

The first indication that the egg coat is an amyloid was in fish and silkmoth oocytes, where it was suggested that the highly stable amyloid structure protected the oocyte under stress conditions [38,39,40]. Our studies of the mouse ZP showed that it has the properties of an amyloid and that all three ZP proteins may contribute as amyloids to the formation of the ZP matrix [41]. Further, an evolutionary analysis of the ZP polymerization domain from ZP3 homologs from fish to human showed that, despite a lack of sequence similarity, there was a striking conservation between all six taxa examined in the regions that were predicted to be amyloidogenic (Figure 2B) [41]. Several of these amyloidogenic amino acid stretches localized to regions predicted to form β-strands based on chicken ZP3 crystal structure including regions important for interactions between ZP subdomains [42]. Comparable regions of amyloidogenic propensity were also found in yeast α-agglutinin suggesting that molecules important for mammalian fertilization may have taken their origins from those involved in yeast mating, an early form of sexual reproduction (Figure 2B) [43]. Other amyloidogenic sites were detected in regions outside of the ZP polymerization domain including the ZP-N repeats which are implicated in mediating interactions with spermatozoa (Figure 2A) [44,45]. In further support that amyloidogenesis may drive ZP matrix formation and function are studies of human ZP proteins. Peptides corresponding to regions of human ZP proteins implicated in a critical β-strand interphase involved in ZP formation assemble into amyloids [46,47]. In all ZP proteins examined, none possess a classic prion-like domain with Q/N-rich regions but instead contain multiple short amino acid stretches with predicted inherent amyloidogenic propensity as determined by the AmylPred2 algorithm [41].

The significance of the ZP/egg coat as an amyloid may be several-fold. As proposed in the fish and silkmoth, the inherent stability of the ZP amyloid would provide protection for the oocyte, especially when surrounded by proteases and other hydrolytic enzymes released from spermatozoa. Since ZP structure is important for later processes in early embryogenesis, amyloids may ensure that the structure remains intact after sperm penetration [48]. We speculate that the amyloidogenic properties of the ZP may also be important for other functions. The presence of three ZP proteins, each with 6–8 predicted amyloidogenic sites, provides the possibility for a multitude of different β-sheet assemblies, in a sense giving the ZP the capacity to modify its structure [49]. Immediately following sperm penetration, a reorganization of the ZP matrix, possibly by the formation of alternate β-sheet structures, might be a mechanism to prevent polyspermy. The ZP as an amyloid may also be significant from the standpoint of how sperm interacts with the ZP. Microcrystals of amyloids show them to be composed of interlocking complementary structures known as steric zippers [49,50]. Based on our studies showing that the mouse sperm AM possesses an amyloid core which includes established ZP binding proteins, this raises the possibility that sperm-ZP binding may involve amyloid–amyloid interactions, possibly by a steric zipper-like mechanism. It is of interest that ZP domains are present in many other proteins that also form fibrils and extracellular matrices but in biological processes unrelated to fertilization. The fact that several of these proteins are also involved in cell–cell interactions suggests that amyloids could be a conserved mechanism for this basic cell biological process [51].

## 6. Epididymal Sperm Maturation

After spermatogenesis, spermatozoa are transported through a long, convoluted tubule known as the epididymis. During epididymal transit, spermatozoa undergo a maturation process and acquire the functions of progressive motility and the ability to fertilize an oocyte. Given that sperm are synthetically inactive, maturation requires the interaction of sperm with proteins secreted into the lumen, in a highly region-dependent manner, by the surrounding epididymal epithelium. In addition to facilitating their maturation, the epididymis must also protect spermatozoa from pathogens including those that ascend into the male tract. However, the mechanisms by which these processes occur are not clear.

**Figure 3:**
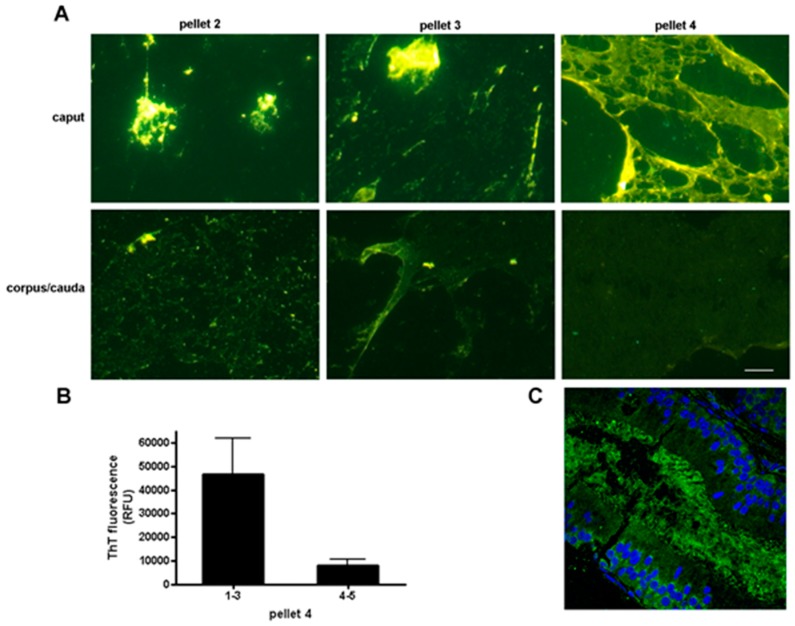
Regional changes in thioflavin staining in the mouse epididymis. (**A**) Particulate material of varying molecular mass was obtained from the caput and corpus-cauda epididymal luminal fluid and stained with thioflavin S. Pellet 2, 5000× *g*; pellet 3, 15,000× *g*; pellet 4, 250,000× *g*. All images were captured with the same exposure times. Bar, 5 µm; (**B**) Thioflavin T fluorescence in pellet 4 obtained from the caput (regions 1–3) and corpus-cauda epididymis (regions 4–5) as determined in a plate assay. Mean + standard error of the mean (SEM) of three experiments; (**C**) Indirect immunofluorescence analysis of amyloid in the caput epididymal lumen using the anti-oligomeric amyloid antibody A11 (green fluorescence) (Muthusubramanian and Cornwall, unpublished observations). Blue, DAPI staining of epididymal epithelial cell nuclei. Modified and reprinted from [52].

We determined that the normal mouse epididymal lumen contains an extracellular matrix with amyloid properties that surrounds the maturing sperm and which may play roles in sperm maturation and protection [52]. The structural properties of the luminal amyloid matrix change along the length of the epididymal tubule transitioning from an abundant amyloid rich in A11 reactive amyloids, suggestive of immature amyloid forms, and strongly stained with thioflavin S (ThS) in the proximal epididymis to a thinner film-like structure with less A11 and ThS staining in the distal epididymis (Figure 3) (Muthusubramanian and Cornwall, unpublished observations) [52]. Whether this change in amyloid properties reflects a matrix that is starting to unwind, or is in fact becoming more highly ordered and unable to efficiently bind these conformation-dependent reagents is not known. However, the changes in amyloid matrix structure parallel changes in epididymal function with the proximal epididymal region carrying out important roles in sperm maturation and distal region serving primarily as a storage site for functionally mature spermatozoa [53].

### Cystatins and the Epididymal Amyloid Matrix

We established that multiple members of the cystatin family 2 of cysteine protease inhibitors contribute to the formation of the luminal amyloid matrix [52,54]. CRES, the defining member of a reproductive subgroup of family 2 cystatins that lack consensus sites for cysteine protease inhibition, and three other CRES family members (CRES2, CRES3, and cystatin E2), along with the prototypical cystatin C colocalize within the amyloid matrix [54]. All CRES family members are highly amyloidogenic in vitro and readily form amyloids with distinct aggregation kinetics [54]. Similarly, cystatin C is an established amyloid [55]. CRES family members and cystatin C do not possess classic prion-like domains but rather are predicted to have multiple amyloidogenic stretches throughout their sequences as determined by the AmylPred2 algorithm [54]. CRES family members are also present in the human epididymis; however, whether they exist as amyloids has not been established [56,57].

Mice lacking the CRES gene show a profound downregulation of other CRES family members, suggesting interrelated functions [54]. Phenotypically, the CRES KO mice develop an age-dependent lysosomal storage disease in the epididymal epithelium, which may reflect an inability of the epithelial cells to degrade aberrant aggregate structures that accumulate in the lumen [58]. Studies are currently ongoing to establish luminal amyloid matrix function, including determining if it serves roles in mediating protein delivery to sperm as part of the maturation process. It is also possible that the amyloid matrix protects spermatozoa from ascending pathogens since CRES, CRES2 and cystatin C have established antimicrobial and/or antiviral activities [56,59,60,61]. Indeed, the CRES family members and cystatin C add to the growing list of amyloidogenic proteins/peptides, including Aβ and α-synuclein that have been shown to possess antimicrobial/antiviral activities and may function as part of the innate immune system [62,63,64].

We hypothesize that interactions between CRES family members and cystatin C are integral for luminal amyloid matrix formation and function, not unlike interactions between bacterial curli family members during the formation of bacterial biofilms [65]. In a broad sense, the epididymal amyloid matrix may be considered as a bacterial biofilm-like structure. Similar to biofilms which integrate bacteria into a community of cells for nutrition and survival, the luminal amyloid matrix may do the same with spermatozoa to facilitate maturation and protection during epididymal transit. 

## 7. Semen Amyloids

During ejaculation, spermatozoa are released in seminal fluid, a combination of fluids originating from the epididymis, prostate and seminal vesicles. Studies by Mϋnch et al. showed that human semen contains amyloid fibrils with the ability to enhance human immunodeficiency virus (HIV) infection in vitro [66]. Specifically, amyloid fibrils form from peptides of prostatic acid phosphatase (SEV1) and semenogelin (SEM), a component of the semen coagulum [66,67]. These SEV1 and SEM amyloids do not appear to be pathological since semen samples were acquired from healthy donors, although their fertility status was not reported. The semen amyloid fibrils capture HIV particles and promote virion-cell attachment and fusion [66,67]. Interestingly, these investigators showed that other amyloidogenic peptides including α-synuclein also enhance HIV-1 infection although with lower efficiency than semen amyloids, suggesting the amyloid fold and not the sequence per se is important for this function [67]. Other investigators demonstrated that SEV1 amyloids can bind bacteria and facilitate their phagocytosis by macrophages [68]. Although not directly exhibiting antimicrobial activity, the SEV1 amyloids may participate, as do many other amyloids, in immune defense.

## 8. Properties of Reproductive Functional Amyloids

### 8.1. Aggregation Properties In Vitro

Proteins that form functional amyloids follow similar aggregation pathways as those that form pathological amyloids, including the development of intermediate oligomeric amyloids which are considered to be the cytotoxic species [69]. One possible means by which functional amyloids do not cause disease is that their assembly may occur under controlled cellular conditions that limit exposure to the oligomeric forms. Of the functional amyloids studied to date, many of the proteins transition quite rapidly from monomeric to mature amyloid forms, possibly as a means to avoid cytotoxic intermediates [70]. Indeed, unlike pathological amyloids such as α-synuclein, which requires days to weeks to form mature amyloid fibrils in vitro, proteins forming functional amyloids often do so within minutes to hours [70,71].

Our results examining the kinetics of CRES family amyloidogenesis are consistent with other proteins that form functional amyloids by showing rapid rates of amyloid formation in vitro. Immediately following dilution from 6M guanidine-HCl into nondenaturing buffer, all CRES family members form amyloids with slightly different structural and aggregation properties [54]. ThT plate assays, negative stain electron microscopy (EM), and dot blot with conformation-dependent antibodies show that while CRES formed fibrils, oligomeric and protofibril forms were also present. In contrast, CRES3 rapidly transitions within minutes to stable polygon, highly ThT reactive structures with little or no oligomeric forms detected [54]. Thus, among the four CRES subgroup proteins examined in our studies, CRES is the least amyloidogenic and CRES3 the most amyloidogenic, with CRES2 and cystatin E2 falling in between these two extremes. Our studies of the CRES family in vitro are consistent with our observations in vivo. In mouse epididymal luminal fluid, CRES is present in both soluble and insoluble fractions while CRES3 is only detected in the insoluble fraction [54]. Studies are currently ongoing to determine if CRES family members exhibit nucleation-dependent kinetics typical of many amyloidogenic proteins and further if they cross-seed and promote nucleation events with each other. Intriguingly, the polygon morphology of CRES3 is strikingly similar to Rnq1, a yeast prion with established roles in nucleation of other amyloidogenic proteins, suggesting that CRES3 may perform similar functions in luminal amyloid matrix assembly [72]. In addition to the rapid kinetics of amyloidogenesis in each CRES family member, interactions between family members and cystatin C may be a mechanism to control and facilitate amyloid matrix assembly in the epididymis. This is similar to coordinated interactions that occur between bacterial curli proteins in biofilm formation [65].

Other reproductive functional amyloids also exhibit fast rates of amyloid formation including Xvelo, the amyloid component of Balbiani bodies, which transitioned into a fibrillar matrix after 12–24 h [5]. Similarly, fibrils were detected in recombinant Rim4 after overnight incubation [15]. Synthetic peptides of prostatic acid phosphatase (PAP) (SEV1) and SEM also assemble into amyloid fibrils following nucleation-dependent elongation. The lag phase to fibril formation was shortened significantly to less than one hour by addition of seminal plasma or buffers that mimicked the semen environment, suggesting that fibril formation rapidly occurs in vivo [73]. Taken together, proteins forming reproductive functional amyloids exhibit rapid rates of amyloid formation which may be a characteristic common to many functional amyloids.

### 8.2. Structural/Biophysical Properties In Vitro

Although the structures of functional and pathological amyloid fibrils and matrices appear similar when observed by electron microscopy and atomic force microscopy, it is possible there are differences at the atomic level. For this reason, solid-state nuclear magnetic resonance (SSNMR) has become the gold standard for studying amyloids atomistically. SSNMR can resolve subtle structural differences between different amyloids and this information can be leveraged to identify structural divergence between pathological and functional amyloids.

To date, only a few functional amyloids have been examined by SSNMR and of these several were from nonmammalian species or were only of specific protein domains [74,75]. The fact that CRES family members average only 14 kDa molecular weight provides a unique opportunity to study the structural and biophysical properties of these full-length mammalian proteins in both their soluble monomeric and functional amyloid states. Preliminary circular dichroism (CD) and SSNMR suggests that CRES3 transitions from a soluble form with mixed secondary structure to an insoluble form rich in β-sheet, a hallmark of amyloid (Do et al., unpublished observations) [76]. These data are similar to that previously reported for both pathological and functional amyloids [77,78]. Furthermore, preliminary analysis of cell constants in X-ray crystallography studies suggests that the CRES crystal contains a trimer in the asymmetric unit (Hewetson et al., unpublished observations) [79]. These studies indicate that CRES has the ability to self-assemble into higher ordered structures as we would expect for an amyloidogenic protein. Additional biophysical and structural studies of the CRES family members will provide important insight for how these structures form and any distinctions they may have from pathological amyloids.

### 8.3. Reproductive Functional Amyloids In Vivo

Of the few functional amyloids identified to date in the reproductive tract, there appears to be several types: (1) individual proteins that utilize prion-like domains for their self-assembly into an amyloid resulting in altered protein function; and (2) proteins that do not possess classic Q/N-rich prion-like domains, but instead contain multiple short amino acid stretches with amyloidogenic propensity and which assemble with several to many other proteins (related family members or unrelated proteins) into complex amyloid matrices with biological functions. We propose that those in the former category might be like yeast prions which often are DNA/RNA binding proteins that use the amyloid fold to mediate protein function, but which also have the potential to elicit profound changes in phenotype and even the inheritance of new traits [8]. Those in the latter category may serve as relatively stable structural scaffolds but perhaps with the ability to modify their structures and/or interactions possibly through the use of different amyloidogenic sites and formation of new β-sheet assemblies. It is likely that, as more functional amyloids are described, there will be variations on these types as well as new categories, as suggested by Xvelo which has a prion-like domain yet seemingly by itself forms the scaffold infrastructure of the Balbiani body. There are several lines of evidence to support the idea that complex amyloid assemblies are likely. First, studies of proteins that have multiple amyloidogenic segments which include Aβ, tau and islet amyloid polypeptide (IAPP), showed that each segment adopted a different amyloid structure (parallel β-sheets versus antiparallel β-sheets) in vitro despite being from the same protein [49]. From these data, it was proposed that amyloid formed from the entire protein could contain more than one type of β-sheet structure or that fibrils may contain β-sheets generated from more than one amyloidogenic site, either of which could result in highly elaborate amyloid matrices [49]. Second, although the formation of amyloid heterofibrils was initially thought to occur only between related proteins, recent studies show that unrelated proteins can cross-seed and form structurally distinct amyloids suggesting that complex amyloid matrices are possible [80,81]. In these studies, cross-seeding was shown between amyloidogenic proteins associated with pathologies as well as those with functional roles. Taken together, interactions between several or many amyloidogenic proteins, each containing multiple amyloidogenic sites, could conceivably result in an extremely sophisticated amyloid structure that may be required for some biological functions as suggested by our studies of the ZP matrix, sperm AM, and the epididymal luminal amyloid matrix. Finally, based on our experience, complex functional amyloid matrices/films such as the sperm AM and epididymal luminal amyloid matrix often require exposure to 70% formic acid, 90% dimethyl sulfoxide (DMSO) or 1–5% SDS to fully reveal their amyloidogenic properties with conformation-dependent reagents [52,54]. Whether this indicates an amyloid matrix that is very highly ordered and/or masked by interacting proteins is not known.

## 9. Reproductive Functional Amyloids May Become Pathological

The commonalities between functional and pathological amyloids prompts the question of whether some disease-associated amyloids may in fact represent functional amyloids that have gone awry. Our studies of a mouse model for the human disease cystatin C cerebral amyloid angiopathy-Icelandic type suggests that in some cases this may be so. Although wildtype cystatin C is amyloidogenic, a single point mutation in the human cystatin C gene (L68Q) results in a highly unstable and aggregation-prone protein that readily forms amyloid in cerebral arteries of affected individuals, causing an early death [82]. Transgenic mice expressing human L68Q cystatin C exhibit profound fertility defects with male mice unable to generate offspring because of poor sperm motility and viability [83]. Our studies suggest that the presence of L68Q cystatin C amyloid alters the structure of the epididymal luminal amyloid matrix such that it becomes cytotoxic. Indeed, luminal fluid isolated from the L68Q mouse epididymis recapitulated the motility/viability defect when added to wildtype sperm [83]. In contrast, L68Q luminal fluid that had undergone high speed centrifugation to remove the luminal amyloid matrix did not. Although not yet determined, the age-dependent development of a lysosomal storage disease in the epididymal epithelium in the CRES KO mouse may also reflect the luminal amyloid matrix that has become pathological as a result of the loss of the CRES family members [58]. 

Together these studies suggest that, under some conditions, functional amyloids can become pathological. This may be due to a change in the equilibrium between monomeric and amyloid forms or quality control mechanisms that have become overwhelmed. Given our studies suggesting that some functional amyloids are formed by interactions between several amyloidogenic proteins, it is also feasible that a loss or alteration in one amyloidogenic protein may result in an abnormal accumulation of its partner(s), which as an orphan amyloid may cause pathologies. Alternatively, as suggested by our studies of the L68Q mice, an alteration in a component of a complex functional amyloid could change its overall structure resulting in cytotoxic properties.

## 10. Summary

As presented herein, functional amyloids play important roles in several reproductive processes including gametogenesis and fertilization. Some functional amyloids are evolutionarily conserved emphasizing the critical role amyloids play in reproduction and that nature favors the amyloid fold for these processes. Further study of reproductive functional amyloids, including their formation and disassembly, will not only provide a better understanding of basic mechanisms of reproduction, but knowledge that could be used to develop new therapies for diseases caused by pathological amyloids.
